# Is the Trained Nurse Barred?

**Published:** 1920-04-24

**Authors:** 


### STATE AID FOR WOMEN WAR-WORKERS.

### Is the Trained Nurse Barred?

It is announced that a large sum of money, amounting probably to over half a million, is to be granted from the Queen's Fund, and likewise from the National Relief Fund, for the purpose of training women war-workers for suitable careers. This is to be administered by the Central Committee on Women's Training and Employment, of which Lady Crewe is Chairman, Miss Mary Macarthur, Honorary Secretary, and Mrs. H. T. Tennant, Honorary Treasurer.

The idea, in its essence, is magnificent. The women of the kingdom offered their services without stint during the war, and it is no exaggeration to say that in their own sphere they did as much to achieve victory as the men-at-arms. Their sacrifices were not confined to toil and treasure. With British pride and without murmuring they gave their sons, their brothers, and their husbands, and wrapped their sorrow in their hearts. Now that the war is over it is fitting that a grateful country should offer some recompense, and, although half a million sounds substantial, in reality it is a very small sum considering the losses that have been patiently endured, and the large number affected.

### The Scope of the Scheme.

At a recent meeting, held at St. Ermin's Hotel, Miss Macarthur outlined the scope of the scheme. It is to be thoroughly practical. Women of talent and attainment may be given professional training. One girl has already been articled to a solicitor. Girls will be trained in medicine, in dispensing, in sanitary and health work, in domestic science, as midwives, teachers, as chefs—not for the tables of the rich, but for hotels, schools, and the like. Short intensive courses for women demobilised from Government offices, whose education was not up to matriculation standard, are to be established, so that they may be fitted to become matrons in schools and hydropathics.

Applications for training may now be made at Employment Exchanges in Great Britain, and Interviewing Committees will sit in London, Birmingham, Leeds, Manchester, Cardiff, and Bristol. These Committees will consist of a representative of the Central Committee to act as chairman, representatives of the local Education Authority, the Women's Training Branch of the Ministry of Labour, and the Employment Department of the Ministry of Labour. The Universities have promised sympathetic co-operation, and on the whole it may be said that in every respect the prospect is bright.

### The Eligibles.

It is to be hoped, however, that Miss Macarthur has not spoken the last word on the subject when she made the sweeping statement that there is no use in sending in an application form unless the applicant is able to show that she is unemployed as the result of the war or that her earning opportunities have diminished. Probably Miss Macarthur meant as a result of the peace, for it was the war that caused their employment. Assuming this to be so, and being a believer in the doctrine of the survival of the fittest, I venture to query the implied suggestion that unemployment, *per se*, or diminished earning capacity, should establish an exclusive claim to participation. Speaking in general terms, I hold it to be true that the war-worker who is now employed possesses greater efficiency than her sister who is unemployed, and that the community, as well as herself, will derive greater benefit from specialised training because of her efficiency. If I am right, the proposal to award a State grant only to the unemployed is both unjust and economically unsound.

Take a probable case. No woman war-worker toiled more arduously, or sacrificed herself more freely, than the Trained Nurse. Nurse Cavell was the apotheosis of sacrifice and devotion. Far be it from my intention to attempt to depreciate the value of the services of the women who acted as clerks in public offices during the war. They discharged a necessary and important duty. But I am convinced that they themselves would be the first to admit that the country's indebtedness to them is as nothing in comparison with that which is the nurse's due.

According to Miss Macarthur, the nurse is barred from any participation. And, to be truthful and practical, the demobilised woman clerk, the V.A.D. who is unemployed, the munition-worker—all of these, and more also, are eligible to become the recipients of a State bounty which, if taken advantage of, will provide them with careers more remunerative than those which nurses can reasonably hope to enjoy.

At the moment this complaint happens to have an extremely practical application. In enumerating some of the careers for which training grants may be available Miss Macarthur specifies Health work. The Minister of Health declines to admit even Trained Nurses as Health Visitors unless they abandon their employment and spend a year at prescribed training institutions. Obviously very few of them can afford to do this. It offers no consolation to the nurse to know that other women have to spend two years in training. The grim fact that confronts her is that she is effectually barricaded. She can no more take a year's special training than she can embark on an indefinite hunger-strike.

Now, I want to put it to Miss Macarthur—is the Trained Nurse who happens to be at her post, and who has borne the burden and heat of the terrible days of war, out of bounds? Is she ineligible to obtain a grant when she needs it most, just because she is earning her living? I have read and re-read this lady's remarks, as reported in *The Times*, in the vain hope of finding a loophole. I regret to have to state that I can find nothing but an additional barricade.

IERNE.

